# Supplementary material for: Splice-Junction-Based Mapping of Alternative Isoforms in the Human Proteome
Source: Cell Rep. Author manuscript; Available in PMC 2020 Jan 15. (PMC6961840; doi:10.1016/j.celrep.2019.11.026)
Supplement: 2 [file NIHMS1546469-supplement-2.zip › DF1/PXD010154/Thyroid/Thyroid_40_UBAC2_QDIDLYIGTADVR.pdf]

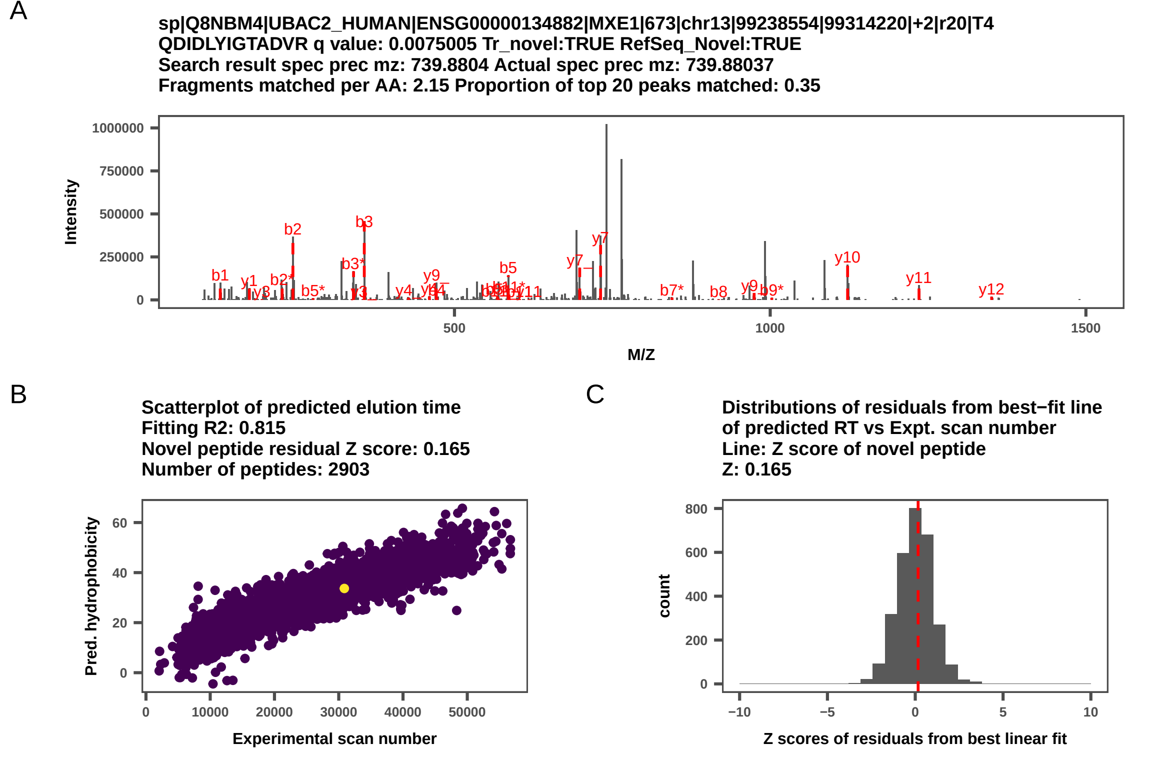
A
sp|Q8NBM4|UBAC2_HUMAN|ENSG00000134882|MXE1|673|chr13|99238554|99314220|+2|r20|T4
QDIDLYIGTADVR q value: 0.0075005 Tr_novel:TRUE RefSeq_Novel:TRUE
Search result spec prec mz: 739.8804 Actual spec prec mz: 739.88037
Fragments matched per AA: 2.15 Proportion of top 20 peaks matched: 0.35
Intensity
M/Z
B
Scatterplot of predicted elution time
Fitting R2: 0.815
Novel peptide residual Z score: 0.165
Number of peptides: 2903
Pred. hydrophobicity
Experimental scan number
C
Distributions of residuals from best−fit line
of predicted RT vs Expt. scan number
Line: Z score of novel peptide
Z: 0.165
count
Z scores of residuals from best linear fit
